# CRISPR-based mutagenesis of lipopolysaccharide biosynthesis genes in *Leptospira interrogans* reveals gene essentiality and confirms the role of an O-antigen polymerase

**DOI:** 10.1038/s41598-026-43869-y

**Published:** 2026-03-13

**Authors:** Luis G. V. Fernandes, Jarlath E. Nally

**Affiliations:** 1 Infectious Bacterial Diseases Research Unit, Agricultural Research Service, United States Department of Agriculture, Ames, IA USA; 2 National Animal Disease Center, 1920 Dayton Avenue, Ames, IA 50010 USA

**Keywords:** *Leptospira*, Leptospirosis, Mutagenesis, Lipopolysaccharide, Mutant, Virulence, Vaccine, Genetics, Microbiology, Molecular biology

## Abstract

Leptospirosis is a worldwide zoonosis caused by pathogenic bacteria of the genus *Leptospira*. Lipopolysaccharide (LPS) is an immunodominant and protective antigen for *Leptospira*, but its biosynthesis remains poorly understood. In this study, we employed CRISPR/Cas9-non-homologous end-joining and CRISPR-Prime Editing to mutate key genes within the *rfb* locus of *L. interrogans*, including those involved in core oligosaccharide assembly, and the biogenesis, polymerization, and ligation of O-antigen. Mutants were successfully generated in LIC11312 (*waaF*, heptosyltransferase II) and LIC12137 (*wcaJ*, undecaprenyl-phosphate glycosyltransferase) but yielded only in-frame deletions suggesting their essentiality. Mutants were also successfully generated in LIC12143, a putative O-antigen polymerase, which exhibited truncated LPS that failed to induce acute leptospirosis in hamsters but retained the ability to colonize kidneys. Mutation of LIC_RS09320, an O-antigen ligase, did not display a change in LPS phenotype. Bacterins prepared with either control wild-type or LIC12143 mutant cells conferred complete homologous protection with sterile immunity, though failed to protect against heterologous challenge. These findings confirm LIC12143 as a functional O-antigen polymerase and underscore the challenges in generating knockout mutants to understand LPS biosynthesis in leptospires.

**Supplementary Information:**

The online version contains supplementary material available at 10.1038/s41598-026-43869-y.

## Introduction

Pathogenic species of *Leptospira* are the causative agents of leptospirosis, a globally distributed bacterial zoonotic disease that affects a broad spectrum of mammalian hosts, including humans^1–3^. Each year, leptospirosis is estimated to cause over one million human infections and almost 60,000 deaths worldwide^4^. Leptospires can infect hosts by penetrating abraded or sodden skin, as well as intact mucosa, subsequently disseminating via the bloodstream to target organs^5^. The clinical manifestations of acute leptospirosis are influenced by the large diversity of pathogenic *Leptospira* species and serovars, the level of exposure, and the host-pathogen interaction^1,6,7^.

Serovar diversity in *Leptospira* spp. is driven by the chemical and structural variability of the sugar moieties that constitute lipopolysaccharides (LPS), an immunodominant and protective antigen. Serovars of *Leptospira* exhibit distinct, though not strictly exclusive, host preferences^2,8^. A correlation between *Leptospira* species and serovars is not always observed; a single species may encompass multiple serovars, while a given serovar can be distributed across different species^2,9^, suggesting horizontal gene transfer of the *rfb* locus, which encodes the majority of enzymes involved in LPS biosynthesis.

*Leptospira* and other spirochetes are diderm bacteria. However, among the phylum Spirochaetota, only *Leptospira* and *Brachyspira* possess lipopolysaccharide (LPS) in their outer membrane^9,10^. Despite its abundance, the structural and functional characteristics of *Leptospira* LPS remain poorly understood. In typical Gram-negative bacteria, smooth LPS is a complex glycolipid that can be structurally divided into three covalently linked parts: lipid A, which serves as the hydrophobic anchor within the membrane, a nonrepeating oligosaccharide core region, and a repeating O-antigen polysaccharide^11^. The core oligosaccharide can be further subdivided into two regions: the inner core, which consists primarily of the eight-carbon sugar Kdo (3-deoxy-D-manno-oct-2-ulosonic acid) and heptose residues, assembled by heptose transferases, and the outer core, which displays greater structural variability across species. LPS is crucial for the viability of most Gram-negative bacteria, with the Kdo2-lipid A moiety being the minimal structural unit required for cell viability in most species^12,13^. However, leptospiral LPS is atypical and it does not exhibit the usual ladder-like pattern observed in other Gram-negative bacteria^14,15^. To date, only two LPS mutants have been recovered following random transposon insertion into *L. interrogans* serovar Manilae, suggesting that the *rfb* locus represents a transposon insertion cold spot, or a locus with genes that are essential for cell viability^15^. One of these mutants displayed a truncated LPS phenotype as a result of transposon insertion into a gene that encodes a protein of unknown function with 11 predicted transmembrane helices.

In most pathogenic *Leptospira* serovars, a conserved gene cluster is responsible for O-polysaccharide biosynthesis through the Wzx/Wzy-dependent pathway^16^, which comprises the integral inner membrane Wzx flippase and the Wzy polymerase. In this pathway, the synthesis of an O-antigen unit begins at the cytoplasmic leaflet of the inner membrane, where it is linked to the lipid carrier undecaprenyl phosphate by undecaprenyl-phosphate glycosyltransferase (wcaJ*)*. This lipid-linked repeat is then translocated to the periplasmic leaflet by the flippase Wzx^17^. In the periplasm, O-antigen subunits are polymerized at the reducing end of the growing chain by the polymerase Wzy^18–20^. Next, the O-antigen is ligated to the lipid A-core oligosaccharide by WaaL, an O-antigen ligase^21,22^, forming a mature LPS molecule that is subsequently transported to the outer membrane via the Lpt pathway^13^.

Given the complexity of the LPS biosynthesis locus and the limited understanding of the roles that individual genes play in the sequential assembly of LPS, particularly due to the scarcity of available LPS mutants, a critical gap in our knowledge remains. To address this, we used CRISPR-Prime Editing (PE)^23^ and CRISPR/Cas9-non-homologous end joining (NHEJ)^24,25^ to mutate genes of the *rfb* locus that facilitate the biogenesis of LPS in *L. interrogans*. These complementary genetic tools have previously been applied in *Leptospira* to generate precise single-nucleotide mutations using CRISPR-Prime Editing, as well as random deletions in target genes resulting from error-prone NHEJ repair following Cas9-induced DNA breaks. The generation of mutants lacking or expressing truncated LPS structures can offer a powerful approach to deepen our understanding of leptospiral biology and virulence, and could pave the way for the generation of serovar-independent bacterins.

## Materials and methods

### Bacterial strains and media

Pathogenic animal isolates of *L. interrogans* serovar Canicola strain LAD-1^23^ and serovar Copenhageni strain R47^26^ were grown in HAN medium^27^ at 29 °C. For agar plates, media was supplemented with 1.2% noble agar (Difco) and 0.4% inactivated rabbit serum. Spectinomycin (40 µg/mL) was used for transconjugant recovery. *E. coli* strain β2163^28^, auxotrophic for diaminopimelic acid (DAP), was grown in Lysogeny Broth (LB, Difco) medium supplemented with DAP (0.3 mM, Sigma).

### Selection of genes in the *rfb* locus for mutation and in silico analysis

Amino acid sequence comparisons were conducted using BLASTp and PSI-BLAST algorithms. Multiple sequence alignments were performed using Clustal Omega (https://www.ebi.ac.uk/jdispatcher/msa/clustalo)^29^. Protein subcellular localization was predicted with PSORTb v3.0.3 (https://www.psort.org/psortb/). Transmembrane helices were identified using TMHMM v2.0 (https://services.healthtech.dtu.dk/services/TMHMM-2.0/) and SMART (https://smart.embl.de/), which also supported orthology and conserved domain analyses. Prediction of three-dimensional structure model of protein was performed by I-TASSER (https://zhanggroup.org/I-TASSER/).

### Plasmid construction and delivery to *Leptospira*

To generate single or double nucleotide deletions within target genes, the CRISPR Prime (PE) strategy was employed. Prime-editing guide RNAs (PEgRNAs) were designed following previously established protocols^23^. Protospacer sequences were selected using CRISPRscan (https://www.crisprscan.org/) based on target loci retrieved from GenBank (https://www.ncbi.nlm.nih.gov/genbank/). PEgRNA cassettes were synthesized by GeneArt (Invitrogen) and subsequently amplified by PCR using PEgRNA-F and PEgRNA-R primers (Supplementary Table 1). The resulting amplicons were used for Gibson Assembly ligation with *Not*I digested pMaOriPE^23^.

To generate random deletion fragments within target genes, the CRISPR/Cas9-NHEJ strategy was employed. PEgRNA cassettes were used as templates for PCR amplification using primers sgRNA-F and PEgRNA-sgRNA-R (Supplementary Table 1). The reverse primer was specifically designed to convert PEgRNAs into sgRNAs by removing the 3′ extension and restoring the terminal thymidine stretch characteristic of the Cas9 handle. The resulting PCR products were ligated via Gibson Assembly into *Xma*I-digested pMaOriNHEJ.Inducible: Cas9 plasmid, as previously described^25^. Gene targets are listed in Table 1, and primers and sequences are described in Supplementary Table 1.

Plasmids of interest were delivered to *Leptospira* cells by conjugation, as previously described^30,31^. Transconjugant colonies were recovered after incubating the HAN plates containing spectinomycin at 29 °C for approximately 14 days. Colonies were randomly selected and inoculated in HAN media for downstream analysis.

### Electrophoresis, immunoblotting and LPS staining

Mid- to late-log phase cells (2-5 × 10⁸/mL) were pelleted (10,000 × *g*, 15 min), washed twice with PBS, and lysed for SDS-PAGE on 4–15% gradient gels (Bio-Rad). Biomolecules were transferred to PVDF membranes (Bio-Rad) via semidry blotting and blocked with SuperBlock (Thermo) for 1 h. Primary antibodies (1:10,000 for rabbit polyclonal anti-Canicola, anti-Icterohaemorrhagiae (National Veterinary Service Laboratories, USDA, Ames, IA), anti-LipL32 and anti-LipL41^32^, 1:5,000 for anti-LigA/B^33^ and monoclonal anti-Canicola (Centre for Veterinary Biologics, USDA, Ames, IA) were diluted in blocking buffer. After 1 h incubation, membranes were washed (PBS-T) and probed with HRP-conjugated secondary antibodies (1:4,000) (Sigma, MO, USA). Detection used Clarity Max ECL (Bio-Rad) and ChemiDoc MP imaging. Lipopolysaccharide was visualized by staining 5 × 10^7^ cell lysates with Pro-Q Emerald 300 (Invitrogen) per manufacturer’s instructions.

### DNA sequencing

Total DNA from mutant and control *L. interrogans* transconjugants were used for PCR of target genes using primers flanking the expected mutation site (Supplementary Table 1). PCR reactions were visualized on 1% agarose gels. Amplicons were then purified, and the final product was used for Sanger DNA sequencing^34,35^ with the same primers used for amplification. Sequences were used for alignment with wild-type sequences by Clustal Omega. Amplicons were also confirmed by Nanopore sequencing (Plasmidsaurus Inc, Arcadia, California).

### Animal ethics statement

All animal experimentation was conducted in accordance with protocols as reviewed and approved by the Animal Care and Use Committee at the National Animal Disease Center, and as approved by USDA Institutional guidelines. Weaned female hamsters were acclimated to the facility a week prior to challenge at 4–5 weeks of age. Animals were monitored daily and always had *ad libitum* access to food and water. Animals were kept in solid-bottom cages containing wood chip bedding and maintained under standard environmental conditions. To support animal welfare, environmental enrichment was provided. All animal experiments described in this manuscript were conducted in accordance with the ARRIVE guidelines.

### Hamster infection experiments

Outbred, recently weaned female Syrian hamsters *(Mesocricetus auratus*, *n* = 5 per group/cage) (Charles River Laboratories, Wilmington, MA) were intraperitoneally inoculated with 10^7^ control or mutant leptospires. A non-infected group inoculated with media only was included as negative controls. Animals were monitored daily for clinical signs of acute leptospirosis, weight, and humanely euthanized upon ≥ 10% weight loss and/or observation of additional clinical signs (blood on paws, nose, or urogenital tract; lethargy), as previously described^36^. On day 3 post-infection, animals were anesthetized using isoflurane and blood samples were collected via retroorbital plexus for quantification of leptospiremia. Animals were monitored after anesthesia until full recovery as assessed by mobility. At designated time points, hamsters were anesthetized using ketamine/xylazine and euthanized by exsanguination. One kidney and one liver lobe were harvested, macerated in 5 mL of HAN medium supplemented with 5-fluorouracil (5-FU), and used to inoculate fresh medium at different dilutions. Cultures were incubated at 37 °C and monitored daily by dark-field microscopy; recovered mutants were confirmed by immunoblotting. Additional sections of kidney and liver were immediately frozen for subsequent quantitative PCR analysis to assess bacterial load.

Approximately 25–75 mg of kidney cortex and liver tissue were homogenized in 500 µL of PBS using a motorized pestle. A volume corresponding to 25 mg of tissue was used for DNA extraction with the Maxwell^®^ RSC PureFood Pathogen Kit, following the manufacturer’s specifications and a final elution volume of 100 µL. Bacterial load in target organs was quantified using a TaqMan-based quantitative PCR assay on a QuantStudio 3 (Thermo Fisher Scientific), targeting the *lipL32* gene as previously described^37^. Samples were run in triplicate and considered negative if no amplification was detected or if amplification resulted in a genome equivalent (Geq)/well below 1.

### Bacterin preparation, hamster vaccination and challenge

Control (full length wild-type LPS) and LIC12143 mutant (truncated LPS) *Leptospira* strain LAD-1 were cultured in HAN medium at 37 °C under agitation until cell densities exceeded 2 × 10^9^/mL. For bacterin preparation, a volume equivalent to 10^9^ cells, including the culture medium and any secreted antigens, was heat-inactivated at 56 °C for 30 min. To confirm the absence of viable leptospires, 10 µL of the inactivated preparation were inoculated into fresh HAN medium and monitored for growth. Inoculation doses were prepared by mixing equal volumes of the heat-inactivated leptospiral preparation with Freund’s complete adjuvant for the primary immunization, or with Freund’s incomplete adjuvant for the booster immunization. Negative controls were prepared by mixing 500 µL of HAN medium with 500 µL of adjuvant. Hamsters (*n* = 5 per group) were immunized subcutaneously with bacterin at a two-week interval and challenged two weeks after the booster dose by intraperitoneal inoculation with 10^6^ of either homologous *L. interrogans* serovar Canicola LAD-1 or heterologous *L. interrogans* serovar Copenhageni strain R47. Animals were monitored daily for clinical signs of acute leptospirosis, weight, and humanely euthanized upon reaching established endpoints. On day 3 post-infection, blood samples were collected, and one kidney and one liver lobe were harvested on endpoints or at the end of the experiment, for culture and bacterial quantification by qPCR as above described.

### ELISA

High-binding ELISA plates (Costar) were coated overnight with 100 µL of heat-inactivated strain LAD-1 (OD_420nm_=0.1). For that, LAD-1 cells cultured at 37 °C were harvested, washed twice with PBS, resuspended in PBS, and heat-inactivated. Plates were washed with PBS-T (0.05% Tween 20) and blocked with SuperBlock (Thermo) for 1 h. Diluted hamster sera were added and incubated for 1 h, followed by three washes. HRP-conjugated anti-hamster IgG, IgG1, IgG2/3, or IgG3 (Southern Biotech, 1:5,000 in SuperBlock) were added and incubated for 1 h. After extensive washing, 100 µL of KPL SuperBlue TMB substrate (SeraCare) was added. Reactions were stopped after 10 min with 100 µL of KPL TMB BlueSTOP, and absorbance was read at 650 nm using a BioTek Synergy Neo2 plate reader (Agilent, Fisher Scientific).

### Statistical analysis

Bacterial loads in liver, kidney, and blood were analyzed independently using GraphPad Prism. Comparisons between control and mutant-infected groups were performed using non-parametric Mann-Whitney test. Data are presented as geometric means with corresponding error values, and statistical significance was defined as *P* ≤ 0.05. ELISA data were compared by two-tailed t-test. Endpoints of animals, either infected with mutant and control strains, or after challenge in vaccination experiments, were compared using the Log-rank (Mantel–Cox) test, with *P* < 0.05 considered statistically significant.

## Results

### Selection of genes in the *rfb* locus for mutation

Based on published data for the *rfb* locus in *Leptospira* spp^16^., genes predicted to encode a Wzx flippase (LIC12135), Wzy O-antigen polymerase (LIC12136), and WcaJ undecaprenyl-phosphate glycosyltransferase (LIC12137) were selected for mutation. A BLASTp search using the annotated *wzy* from *L. noguchii* serovar Panama^38^ identified LIC12143 as the corresponding homolog in *L. interrogans* serovar Copenhageni strain Fiocruz L1-130, instead of LIC12136, whose ortholog is annotated as a DUF1229 domain-containing protein in *L. noguchii*. BLASTp analysis of annotated O-antigen ligases from *L. noguchii* (LEP1GSC059_RS19925, _RS19550, _RS19050, and _RS04330) identified corresponding homologs in *L. interrogans* Fiocruz L1-130: LIC11753, LIC_RS09320, LIC11923, and LIC11092. Notably, BLASTp searches using O-antigen ligases from *Klebsiella pneumoniae* (YP_001337618.1), *Salmonella enterica* (YP_152678.1), and *E. coli* (NP_418079.1) also matched these same four genes, reinforcing their putative roles as O-antigen ligase. For this work, we selected LIC11753 and LIC_RS09320 for mutagenesis, as they were previously implicated to act as an O-antigen ligase^38,39^.

In addition, to target the oligosaccharide core of the lipopolysaccharide, the gene encoding WaaF lipopolysaccharide heptosyltransferase II (LIC11312) was selected. All target genes were selected from the genome sequence of *L. interrogans* serovar Copenhageni strain Fiocruz L1-130^40^ for guide RNA design (Table 1) and constructs were used for genetic manipulation of orthologs in *L. interrogans* serovar Canicola strain LAD-1.

**Table 1:** Selected gene targets for mutagenesis.

Gene ID	Annotation	Putative role
LIC11312 (LIC_RS06750)	*waaF*	heptosyltransferase II
LIC12137 (LIC_RS10910)	*wcaJ*	undecaprenyl-phosphate glycosyltransferase
LIC12135 (LIC_RS10900)	*wzx*	O-antigen flippase
LIC12136 (LIC_RS10905)	wzy	O-antigen polymerase
LIC12143 (LIC_RS10945)	wzy	O-antigen polymerase
LIC11753 (LIC_RS08945)	*waaL*	O-antigen ligase
LIC_RS09320	*waaL*	O-antigen ligase

### Mutant recovery and validation

**Fig. 1:**
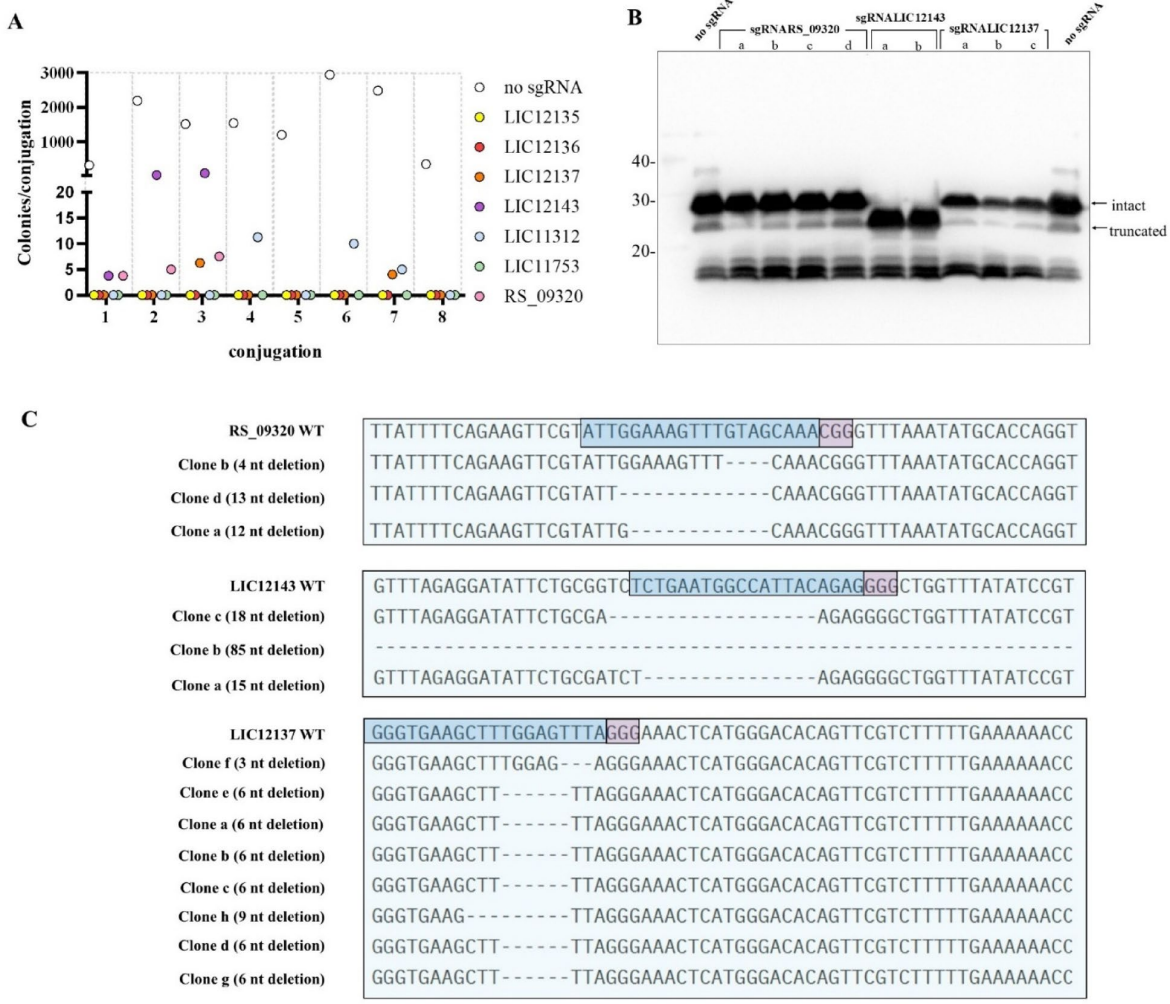
Transconjugant recovery and phenotype and sequencing evaluation of mutants from selected targets. (**A**) Transconjugants of *L. interrogans* serovar Canicola strain LAD-1 were recovered following conjugation with *E. coli* harboring either the empty plasmid pMaOriNHEJ.Inducible: Cas9 or plasmids containing sgRNA cassettes targeting LPS-related genes. Recovered colonies were cultured in liquid medium and subsequently analyzed by immunoblotting using anti-Canicola polyclonal antiserum (**B**). Selected clones were also subjected to PCR amplification of the target loci, followed by sequencing (**C**). The wild-type sequence from *L. interrogans* strain Fiocruz L1-130 is shown, with the protospacer region highlighted in blue and the PAM (5′-NGG-3′) in pink.

Plasmids pMaOriNHEJ.Inducible: Cas9, either empty or containing sgRNA cassettes targeting selected genes, were delivered to *L. interrogans* serovar Canicola strain LAD-1 via conjugation. Across eight independent experiments, mutant transconjugants were only recovered for LIC11312, LIC12137, LIC12143, and LIC_RS09320 (Fig. 1A). Transconjugants were randomly selected and evaluated by immunoblotting with anti-Canicola antiserum (Fig. 1B) and gene sequencing (Fig. 1C). Mutation of LIC12143 resulted in a phenotypic change to the expression of LPS. In contrast, and despite multiple confirmed frameshift mutations in LIC_RS09320 (Fig. 1C), immunoblot analysis revealed no discernible phenotypic alterations for LPS. Of note, only in-frame deletions of 3, 6, or 9 nucleotides were observed after mutagenesis of LIC12137 suggesting it is essential for leptospiral viability.

LIC12143 mutants exhibited a lower molecular mass LPS profile, as revealed by immunoblotting (Fig. 1B), suggesting defective O-antigen polymerization. Transconjugants recovered from the experiments harbored a diverse range of deletions (Fig. 1C), including variants with up to 112 nucleotides deletion (data not shown). Notably, even the in-frame deletions observed in clones “a” and “c” were sufficient to alter the phenotype of LPS, indicating that critical amino acid residues were likely disrupted.

To further investigate targets where no mutations or only in-frame deletions had been recovered, we employed a CRISPR-PE strategy to introduce PAM-disruptive frameshift deletions of 1 or 2 nucleotides in LIC12137, LIC12135, LIC12136 and LIC11753. As with CRISPR/Cas9-NHEJ, mutant colonies could not be recovered; all sequenced clones retained wild-type sequences (not shown) suggesting strong selective pressure against disruptive mutations in these genes.

### Disruption in the heptosyltransferase II (LIC11312) gene is not tolerated by *Leptospira* cells

**Fig. 2:**
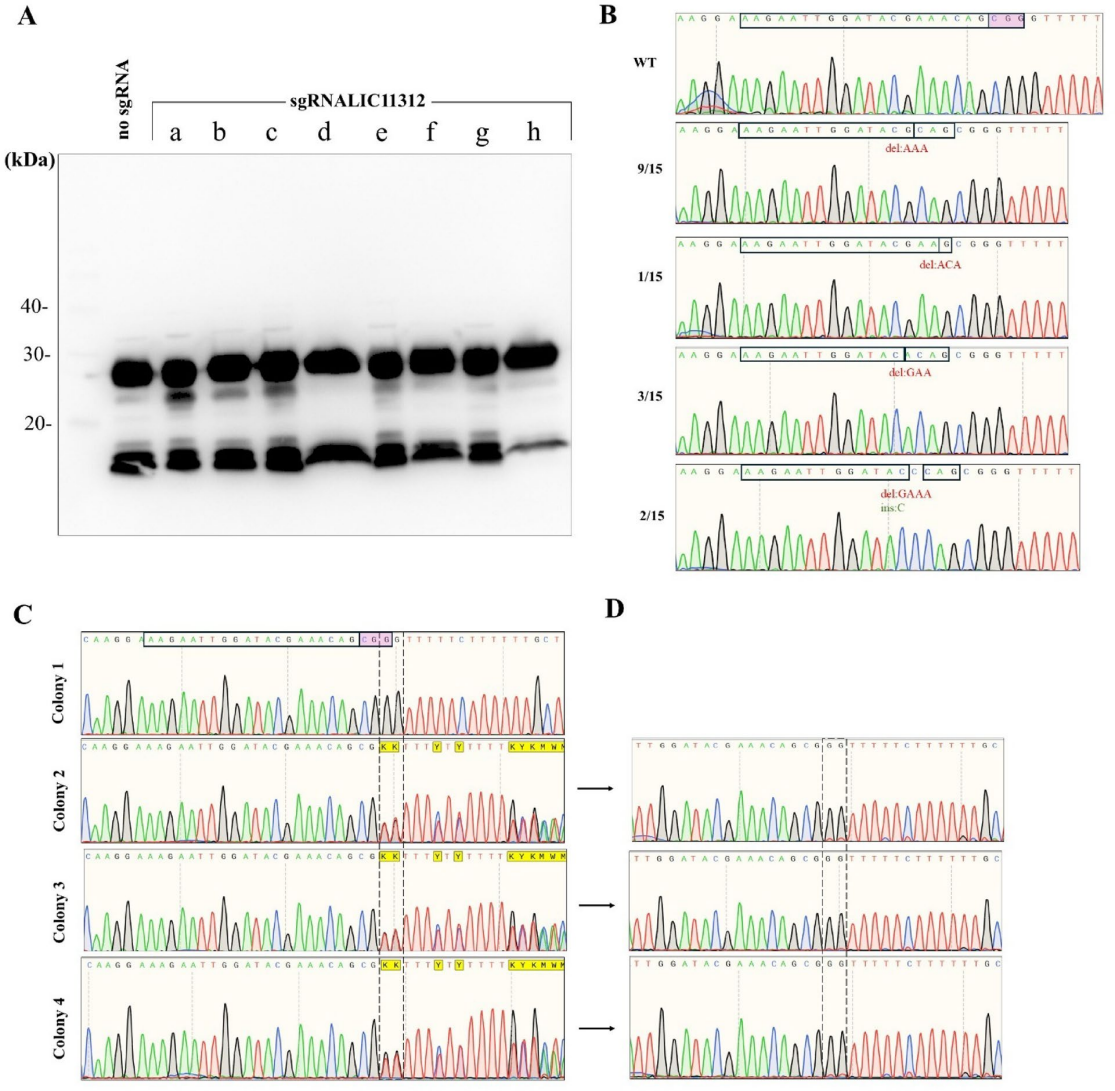
Evaluation of heptosyltransferase II (LIC11312) mutants. (**A**) Selected transconjugant colonies obtained via the inducible CRISPR/Cas9-NHEJ strategy were analyzed by immunoblotting using anti-Canicola polyclonal antiserum. These, along with additional colonies (*n* = 15), were subjected to PCR amplification of the LIC11312 locus, followed by Sanger sequencing which identified in-frame mutations (**B**). The protospacer region is highlighted, with the adjacent motif 5′-NGG-3′ indicated in pink, and each mutation is shown. Transconjugants recovered after conjugation with *E. coli* carrying the pMaOriPE plasmid (CRISPR-Prime editing), either alone or with a PEgRNA cassette targeting LIC11312 for a two-nucleotide deletion, were sequenced immediately after colony isolation (**C**) and again following growth in liquid medium supplemented with spectinomycin (**D**).

Disruption of LIC11312 using the CRISPR/Cas9-NHEJ strategy yielded mutant clones across distinct conjugation experiments (Fig. 1A). However, immunoblot analysis of selected clones revealed no detectable alterations in LPS structure (Fig. 2A). Interestingly, sequencing analysis of 15 mutant clones revealed a consistent pattern, where all exhibited either in frame deletions of three nucleotides or a combination of a four-nucleotide deletion with a single-nucleotide insertion, effectively restoring the open reading frame (Fig. 2B). The inability to recover frameshift mutants suggests that LIC11312 may be essential to cell viability.

The delivery of CRISPR-PE plasmids designed to create a two nucleotide-deletion in LIC11312 resulted in the recovery of several colonies, since CRISPR-PE is a DSB-free technique that does not impact cell survival. Sixteen colonies were randomly selected from agar plates, and leptospiral cells released directly from the medium were used as templates for PCR amplification to assess mutation status. Figure 2C shows representative chromatograms from one fully wild-type colony (Colony 1), and three transconjugant colonies (Colonies 2–4) which exhibited mixed populations of mutant and wild-type sequences as indicated by overlapping peaks immediately downstream of the mutation site. However, when mixed populations were inoculated into HAN medium supplemented with spectinomycin, sequencing of mid-log phase cultures revealed complete loss of the mutation (Fig. 2D). Furthermore, upon replating and sequencing of individual colonies derived from these cultures, only wild-type sequences were recovered, indicating that cells lacking functional LIC11312 are not viable.

### Functional evaluation of O-antigen polymerase LIC12143 and knockout mutants

**Fig. 3:**
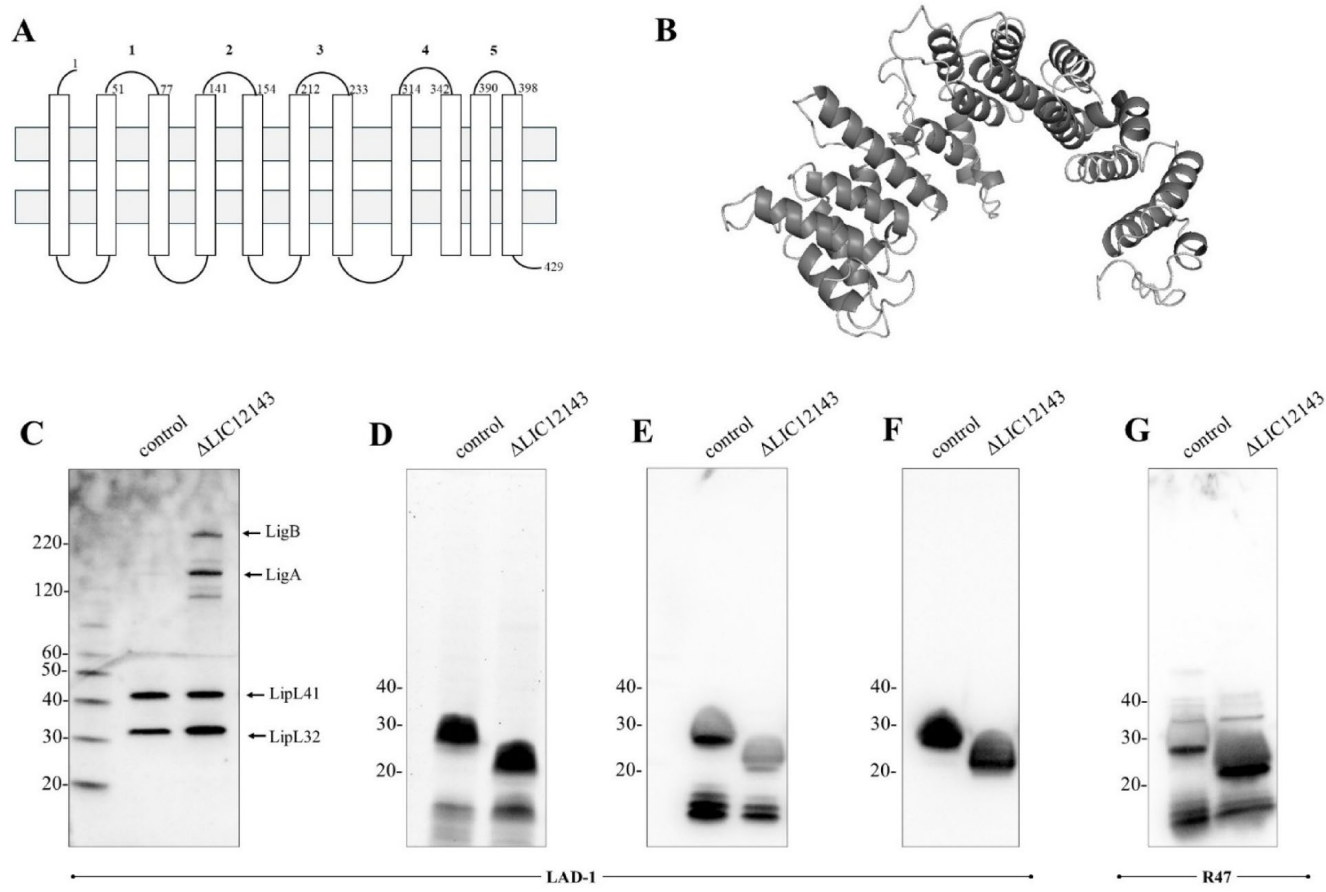
LIC12143 protein exhibits O-antigen polymerase activity in distinct serovars of *Leptospira*. (**A**) The predicted topological model of the LIC12143 protein indicates the presence of 11 transmembrane helices, five periplasmic loops, and cytoplasmic membrane association. (**B**) A three-dimensional structural model of LIC12143 was generated using the I-TASSER server based on its amino acid sequence. (**C**) Control and LIC12143 knockout (*ΔLIC12143*) *L. interrogans* serovar Canicola LAD-1 cells were analyzed by immunoblotting using anti-LipL32, anti-LipL41 (1:10,000), and anti-LigAB (1:4,000) antisera. (**D**) LPS content and molecular size were assessed by Pro-Q Emerald 300 staining and further evaluated by immunoblotting with anti-Canicola polyclonal (**E**) and monoclonal (**F**) antibodies. The LIC12143 gene was also disrupted in *L. interrogans* serovar Copenhageni strain R47 (**G**), and both wild-type and mutant cells were analyzed by immunoblotting using anti-Icterohaemorrhagiae serum.

Bacterial O-antigen polymerases are typically highly hydrophobic and contain at least 11 predicted transmembrane domains^41^. In silico analysis of the LIC12143 predicted protein sequence revealed the presence of 11 transmembrane helices spanning the cytoplasmic membrane (Fig. 3A). Orthology analysis using the SMART webserver revealed homology to the O-antigen polymerase Wzy found in other bacterial species. The LIC12143 protein contains five predicted periplasmic loops, which may mediate interaction with the monomeric O-antigen substrate. Interestingly, in frame deletions in mutants “a” and “c” (Fig. 1C) disrupted amino acid residues within the second periplasmic loop, suggesting its functional relevance. 3D modeling of LIC12143 protein confirms the predominance of helices and loops.

Immunoblot analysis comparing the LIC12143 mutant to the LAD-1 WT control strain revealed upregulation of the well-characterized virulence factors LigA and LigB (Fig. 3C), suggesting a potential compensatory or regulatory response linked to the disruption of LIC12143. Whole-cell lysates were also used for LPS staining (Fig. 3D) and immunoblots with polyclonal (Fig. 3E) and monoclonal (Fig. 3F) anti-Canicola antibodies. As expected, monoclonal anti-Canicola antibodies selectively recognized the O-antigen moiety, regardless of whether it was presented in full-length or truncated LPS molecules. Despite observable differences in LPS size, no variation in MAT titers was detected between control and mutant strains when tested with reference anti-Canicola serum (data not shown). Furthermore, when control and mutant strains were exposed to normal hamster serum, no statistically significant differences in complement susceptibility were observed (data not shown). Disruption of the LIC12143 ortholog in *L. interrogans* serovar Copenhageni strain R47 resulted in a comparable phenotype, as evidenced by immunoblot analysis using anti-Icterohaemorrhagiae polyclonal antibodies (Fig. 3G). This consistency across different serovars reinforces the conserved role of LIC12143 as an O-antigen polymerase. Of note, we did not observe upregulation of LigA or LigB in strain R47 (data not shown).

### LIC12143 mutant is unable to cause acute disease in the hamster model

**Fig. 4:**
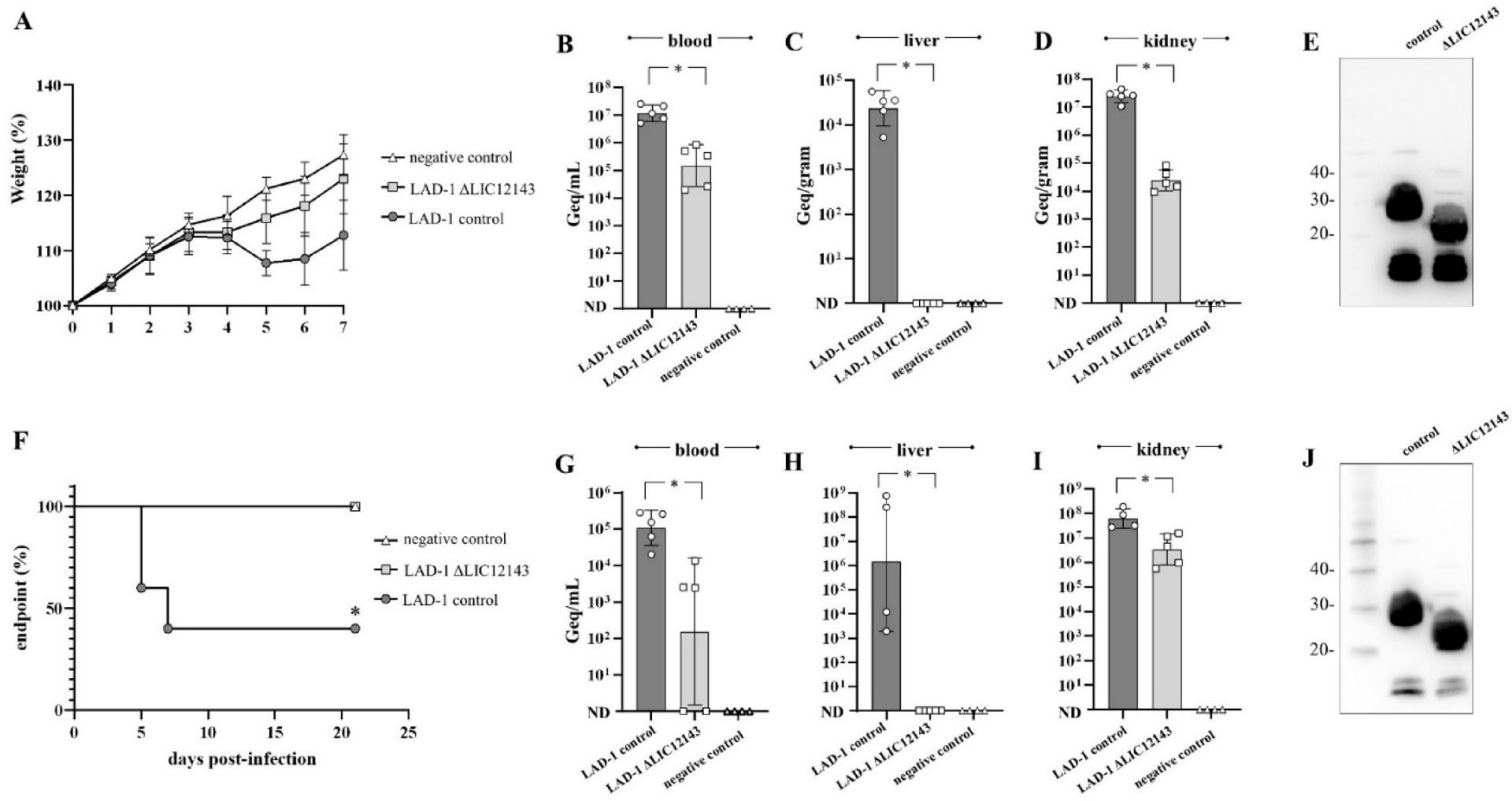
LIC12143 mutant fails to induce acute leptospirosis in hamsters but retains renal colonization capacity. (**A**) Hamsters (*n* = 5) were intraperitoneally infected with 10⁷ cells of either wild-type or LIC12143 mutant *L. interrogans* serovar Canicola LAD-1. Animals were monitored daily for clinical signs of leptospirosis and changes in body weight. Negative control animals received medium only. (**B**) On day 3 post-infection, blood samples were collected via retroorbital plexus for bacterial quantification, expressed as genome equivalents (Geq) per mL. (**C**, **D**) On day 7, all animals were humanely euthanized. Liver (**C**) and kidney (**D**) tissues were harvested for bacterial burden analysis by qPCR. (**E**) Kidney macerates were inoculated into HAN medium supplemented with 5-fluorouracil (5-FU) for bacterial isolation. Isolates were phenotypically confirmed by immunoblotting using anti-Canicola polyclonal antiserum. (**F**) Mutants isolated from the initial experiment were used to re-infect a second cohort of hamsters (*n* = 5), which were monitored daily and euthanized upon reaching humane endpoint criteria. (**G**–**I**) Bacterial burden was assessed by qPCR in blood on day 3 (**G**), and in liver (**H**) and kidney (**I**) at endpoint or at the conclusion of the experiment. (**J**) Kidney-derived isolates were re-validated by immunoblotting. Endpoints of animals were compared using the Log-rank (Mantel–Cox) test. For bacterial burden analysis, comparisons between control and mutant-infected groups were performed using non-parametric Mann-Whitney test (*P ≤ 0.05).

Passage-synchronized *L. interrogans* LAD-1 control cells (harboring empty pMaOriNHEJ.Inducible: Cas9) or LIC12143 mutants were used to intraperitoneally infect hamsters. Animals were monitored daily for signs of acute leptospirosis. Although weight loss and prostration occurred only in the control group (Fig. 4A), all animals recovered fully, and none met endpoint criteria. Consequently, all animals were euthanized on day 7 to allow direct comparison of bacterial burden in target organs and recovery of low-passage cells for subsequent infection experiments. Animals infected with mutant cells exhibited significantly reduced bacteremia on day 3 (Fig. 4B), accompanied by undetectable bacterial burden in the liver (Fig. 4C) and lower levels in the kidney (Fig. 4D) compared to those infected with control LAD-1. Viable leptospires were recovered from the kidneys of all infected animals. Immunoblotting of these isolates confirmed that the mutant strain expressing a truncated LPS retained the ability to colonize renal tissue (Fig. 4E).

Recovered low-passage control and mutant cells were used to re-infect hamsters for phenotype confirmation. All animals infected with control cells displayed signs of acute leptospirosis, with 3 out of 5 reaching endpoint criteria and requiring euthanasia (*P* < 0.05). The remaining two exhibited weight loss but recovered and survived until the end of the experiment (Fig. 4F). In contrast, non-infected negative controls and animals inoculated with the LIC12143 mutant showed no signs of leptospirosis, and none met endpoint criteria. In agreement with the first experiment, significantly lower bacterial burden was found in blood on day 3 (Fig. 4G). Higher leptospiral DNA was detected in the liver of acutely ill animals that reached endpoint (Fig. 4H), while kidney bacterial burden was significantly lower in those infected with mutant strains (Fig. 4I). Phenotypes of control and mutant cells recovered from kidneys were confirmed by immunoblotting (Fig. 4J).

### Vaccination with LPS mutant-derived bacterin only provides homologous protection

**Fig. 5:**
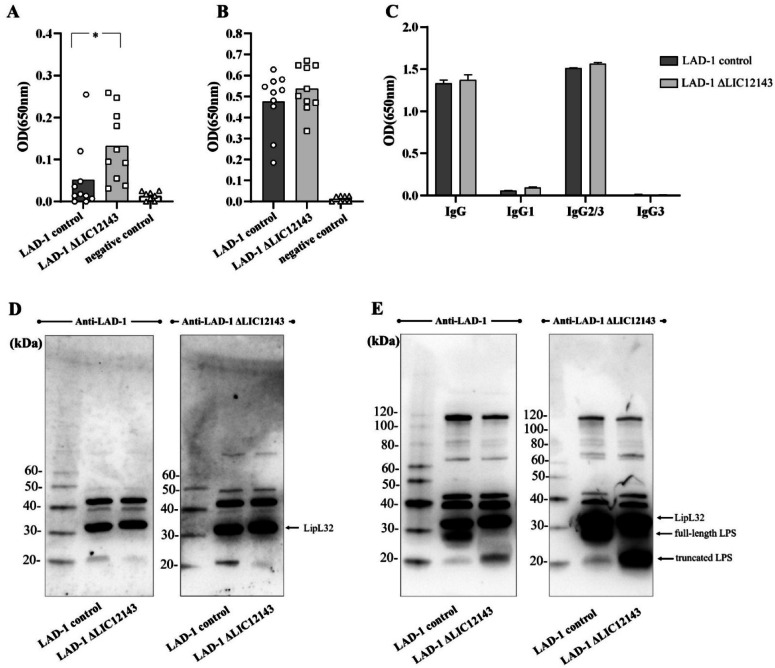
Humoral immune response elicited by control (wild type) and mutant (LIC12143 knockout) bacterins. (**A**, **B**) Individual IgG responses against whole-cell lysates of *L. interrogans* LAD-1 were evaluated by ELISA following prime (**A**) and booster (**B**) immunizations with either control or LIC12143 mutant bacterins. Hamster sera were tested at a 1:800 dilution. (**C**) Isotype profiling was performed using pooled sera from the booster injection (*n* = 10) at 1:800 dilution. LAD-1-coated plates were incubated with sera, followed by horseradish peroxidase-conjugated secondary antibodies specific for total IgG, IgG1, IgG2/3, or IgG3. (**D**, **E**) Antibody reactivity was further assessed by immunoblotting using pooled sera after primary (**D**, 1:500 dilution) and booster (**E**, 1:2,000 dilution) immunizations against lysates from both control and mutant strains. Membranes were exposed for 15 s each. Negative control animals were injected with medium only. ELISA data were compared by two-tailed t-test (*P ≤ 0.05).

Control LAD-1 and LIC12143 mutant were used to prepare whole-cell bacterins for hamster immunization. Sera was collected 2 weeks after primary and booster immunizations, and total IgG antibodies against WT LAD-1 lysates were evaluated by ELISA. Mutant bacterin elicited significantly higher IgG levels following the first immunization (Fig. 5A) in comparison to the control LAD-1 bacterin. The tendency of higher reactivity was also observed after booster injection, although not statistically significant (Fig. 5B). No reactivity against LAD-1 lysate was observed upon incubation with sera from negative control animals (Fig. 5A and B). Pooled sera from each group were used to evaluate IgG isotypes. As shown in Fig. 5C, high reactivity was observed only with anti-hamster IgG2/3 secondary antibodies, in contrast to the low or no reactivity detected for IgG1 and IgG3, respectively. These results indicate that IgG2 was the predominant isotype elicited by immunization.

A prominent anti-LPS IgG response was only observed following the booster injection (Fig. 5D and E). As expected, a strong LipL32-reactive band was already detectable after the first immunization (Fig. 5D). Notably, antibodies raised against the full-length LPS were able to recognize both the full-length and truncated forms, and conversely, antibodies generated against the truncated form also recognized both variants. Additional reactive bands were detected following immunization with both control and mutant bacterin; however, none appeared to be exclusive to either preparation. No differences in MAT titers were observed in the immune sera from WT- or mutant-immunized animals when tested against strain LAD-1 (not shown).

**Fig. 6:**
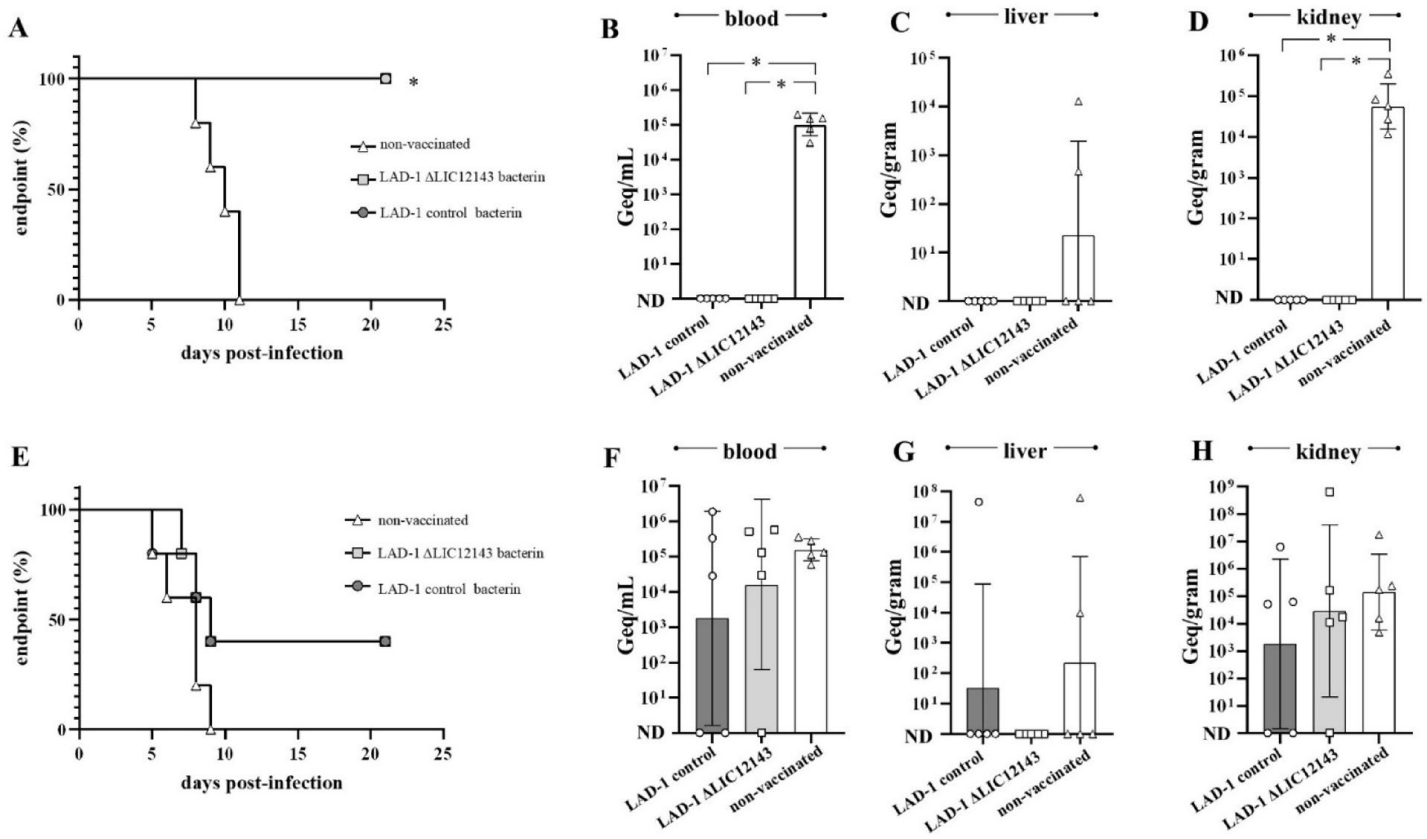
Control and LIC12143 mutant bacterins confer homologous but not heterologous protection. (**A**–**D**) Hamsters (*n* = 5) were vaccinated with two doses of either wild-type or LIC12143 mutant bacterin, or received medium only (negative control), and subsequently challenged intraperitoneally with 10⁶ cells of wild-type *L. interrogans* serovar Canicola strain LAD-1 (homologous challenge). Animals were monitored daily for clinical signs of acute leptospirosis and euthanized upon reaching humane endpoint criteria. Bacterial burden was assessed by qPCR in blood on day 3 (**B**), and in liver (**C**) and kidney (**D**) at endpoint or at the conclusion of the experiment. (**E**–**H**) A parallel cohort (*n* = 5) was challenged with *L. interrogans* serovar Copenhageni strain R47 (heterologous challenge) following the same vaccination protocol. Bacterial burden was similarly evaluated in blood on day 3 (**F**), and in liver (**G**) and kidney (**H**) at endpoint or experiment completion. Endpoints of animals were compared using the Log-rank (Mantel–Cox) test. For bacterial burden analysis, comparisons between control and mutant-infected groups were performed using non-parametric Mann-Whitney test (*P ≤ 0.05).

Following homologous challenge with wild-type *L. interrogans* LAD-1, all animals immunized with either bacterin survived the infection and showed no signs of acute leptospirosis (Fig. 6A), consistent with the absence of detectable leptospires in blood samples collected on day 3 (Fig. 6B). Furthermore, by the end of the experiment, no leptospires could be detected or cultured from kidney tissues (Fig. 6D). In contrast, all non-vaccinated control animals displayed signs of acute leptospirosis and reached endpoint from day 8 to 11, with bacterial loads being detected in their blood, liver and kidney tissues (Fig. 6B–D).

Following heterologous challenge with *L. interrogans* serovar Copenhageni strain R47, only 40% of animals (2/5) survived, irrespective of the bacterin administered (Fig. 6E). No statistically significant difference was observed compared to the non-vaccinated group, whose animals met endpoint criteria from day 5 to 9. Two animals from the control bacterin group and one from the mutant bacterin group completely cleared the infection, as evidenced by the absence of detectable bacterial load in blood, kidney, and liver from these animals (Fig. 6F–H). However, bacterial loads in control and mutant bacterin-vaccinated animals did not differ significantly from those in the non-vaccinated group.

## Discussion

The genus *Leptospira* comprises over 250 serovars grouped into > 26 serogroups, based on the differential serological reactivity of their lipopolysaccharides (LPS) with reference sera^1,42^. Variation in the lengths and sugar compositions of O-antigen subunits across strains form the basis for serotyping, and some species may include multiple serovars, and certain serovars may be shared among distinct species^2^. Despite some variation in the lipid A portion of lipopolysaccharide (LPS) across species, this component remains conserved within individual species, irrespective of serovar classification^9,43^.

LPS is essential for the viability of most Gram-negative bacteria, with the Kdo₂-lipid A moiety representing the minimal structural unit required for growth^12^. Thus, in theory, disruption of the inner and/or outer core assembly of LPS is possible without compromising cell viability. WaaF (heptosyltransferase II) adds the second heptose residue to the Kdo_2_ moiety of the LPS inner core, and mutants for this target have been recovered and validated for *E. coli*^44^. In *E. coli*, the authors observed that deletions leading to the accumulation of O-antigen intermediates frequently resulted in defective cell morphology and lysis. This included mutations in genes encoding the O-antigen flippase, ligase, and *waaF*. Because LPS and peptidoglycan (PG) biosynthesis pathways share the same initial lipid carrier, undecaprenyl phosphate (Und-P), the accumulation of non-functional intermediates sequesters Und-P and impairs its recycling, thereby also disrupting PG synthesis.

The recent canine isolate *L. interrogans* serovar Canicola strain LAD-1 was previously shown to be receptive to genetic manipulation, yielding robust numbers of transconjugant colonies^25^. Attempts to disrupt the *waaF* gene in strain LAD-1 consistently failed to yield viable frameshift knockout mutants. Although the mutated sequence with 2-nt deletion was initially detected in transconjugant populations when CRISPR-PE was employed, it was never recovered in subsequent passages, suggesting that the mutation impairs bacterial growth or viability. This suggests that *waaF* may exert pleiotropic roles in leptospiral cells or that, as observed in *E. coli*, the accumulation of dead-end Und-P intermediates could impair PG or other glycans synthesis, which could play a more drastic role in leptospiral cell viability.

Among all mutagenesis targets in this study, frameshift mutations were identified only in LIC_RS09320 (putative O-antigen ligase) and LIC12143 (putative O-antigen polymerase). However, a discernible phenotype was only observed in the LIC12143 mutant, suggesting that LIC_RS09320 may not function as a canonical O-antigen ligase, or that its role is masked by a functional redundancy of other putative O-antigen ligases. Interestingly, colonies for the O-antigen ligase LIC11753 were never recovered during multiple conjugation experiments. Of note, O-antigen ligase mutants were previously obtained in *Salmonella enterica* serovar Typhimurium^45^, *E. coli*^44^, *Pseudomonas aeruginosa*^21^, and *Helicobacter pylori*^46^.

LIC12143 in *L. interrogans* strain Fiocruz L1-130 was originally annotated to encode an integral membrane protein. Notably, its ortholog in *L. noguchii* is annotated as the Wzy O-antigen polymerase^38^, consistent with orthology, structural, and subcellular localization analyses. Although Wzy mediates the formation of a typical glycosidic bond between O-antigen subunits, it shares no homology to known glycosyltransferases^41^. Inactivation of an O-antigen polymerase is expected to result in a LPS molecule containing only a single repeat unit of the O-antigen, resulting in a shorter, truncated LPS phenotype. Here we show that LIC12143 mutants exhibited a shortened LPS phenotype, which did not compromise agglutination titers or serum resistance; polyclonal and monoclonal anti-Canicola antibodies recognized both intact (WT) and truncated LPS indicating that O-antigens were conserved. Interestingly, the virulence factors LigA and LigB were upregulated in the LIC12143 mutant which is favorable for its use as a bacterin vaccine. Truncation of LPS in *Shigella flexneri* alters the surface exposure and activity of the virulence factor IcsA^47,48^.

Thus far, three mutant strains of *Leptospira* with truncated LPS have been characterized. One strain, designated M895, was constructed by Murray et al.^15^ who used transposon mutagenesis in *L. interrogans* serovar Manilae to identify an insertion within a gene that coded for a product with no predicted function within the *rfb* locus; the mutated gene was orthologous to LA1641 gene in *L. interrogans* serovar Lai^15^ which shares a 100% identity to LIC12143; based on the current analysis, this gene would now be annotated as *wzy*. The two other mutants were spontaneous variants of *L. interrogans* serovar Pomona selected after incubation in homologous antisera^49^. Both mutants contain the insertion element IS1501, and share a disruption in OrfP35, which corresponds to LIC12144, located immediately upstream of LIC12143, suggesting that a polar effect might have occurred.

The *L. interrogans* Manilae mutant M895 failed to induce acute lethal disease in the hamster model of leptospirosis. Neither could mutants be recovered from kidney or liver of inoculated animals, suggesting that the mutant was effectively cleared from target organs and unable to establish persistent infection^15^. Similarly, we report the same mutation in *L. interrogans* serovar Canicola strain LAD-1 yielded attenuated mutants incapable of inducing acute disease in hamsters, with markedly reduced bacterial loads in blood, kidney, and liver. However, in contrast to the findings reported by Murray et al.^15^, the mutant with truncated LPS in the serovar Canicola background remained culturable from the liver and kidney of infected animals, and its phenotype was stably maintained. This discrepancy may be attributed to strain-specific variation, as previously demonstrated in studies silencing both LigA and LigB; knockdown mutants in *L. interrogans* serovar Manilae were avirulent and undetectable in target organs^50^, whereas similar knockdown mutants in serovar Copenhageni, despite being attenuated, remained recoverable from kidney and liver^51^. Nevertheless, the underlying mechanism of attenuation due to shorter LPS remains unknown, since no differences in growth kinetics or complement susceptibility were observed between control and mutant strains.

The *rfb* locus is highly variable, spanning only ~ 4 kb in some strains but exceeding 100 kb in others^16^. Whole-genome analyses of different serovars within the same species revealed that most genes encoding LPS biosynthesis enzymes exhibit clear signatures of horizontal gene transfer^38,52^. In this sense, conservation of the O-antigen polymerase LIC12143 correlates more strongly with serovar identity than with species designation. For example, several *L. interrogans* serovars, including Lai, Copenhageni, Icterohaemorrhagiae, Pyrogenes, and Manilae, harbor a highly conserved LIC12143 ortholog, whereas serovar Hardjo does not. This absence mirrors the situation in *L. borgpetersenii*, where a LIC12143 ortholog is present in serovars Ballum, Tarassovi, Kenya, Ceylonica, and Castellonis, but absent from Hardjo. Nieves et al.^38^ also noted that whenever *wzy* was present, the associated flippase *wzx* was likewise conserved, with a few exceptions, including serovars Hardjo and Guaricura, which encode only the latter.

The host’s adaptive immune response to leptospirosis is predominantly humoral, with protective agglutinating antibodies generated during infection primarily targeting leptospiral LPS, the main antigenic determinant of *Leptospira* spp. As a result, bacterins provide only short-term and serovar-specific protection, despite evidence that protein antigens play a role in mediating protective immune responses^53–55^. Therefore, the prospect of generating novel strains with modified, truncated, or even absent variable regions of the LPS, complemented with increased expression of virulence factors, is highly appealing, especially in light of recent advances in the genetic manipulation of *Leptospira* spp. which now allow editing at single-nucleotide resolution.

The observed upregulation of the virulence factors LigA and LigB, resulting from the mutation in the O-antigen polymerase gene, led us to investigate the immunogenic potential of the mutant strain as a candidate bacterin. Srikram et al.^54^ previously assessed the M895 mutant of *L. interrogans* serovar Manilae as a single-dose live attenuated vaccine in a homologous challenge model. However, the M895 mutant conferred inferior protection compared to the control wild-type bacterin. Here, we observed that complete homologous protection was observed after vaccination with control and mutant bacterin, following two vaccine doses. Antibodies against the full-length LPS could recognize the cognate molecule and also its truncated form, and conversely, antibodies against the shorter LPS recognized both forms. These results align with our observations that WT and mutants had similar titers against anti-Canicola antibodies, and comparable reactivity with polyclonal and monoclonal antibodies between the two immunized groups. As a result, both bacterins displayed an insufficient protective profile following heterologous challenge.

In conclusion, our findings reveal an unexpected essentiality of genes involved in LPS assembly and biosynthesis, underscoring the complexity of the *rfb* locus in *Leptospira*. This essentiality could be due to pleiotropic effects exerted by the targeted genes, or alternatively, from a secondary phenomenon involving the accumulation of dead-end Und-P intermediates, affecting PG biosynthesis. The role of LIC12143 as an O-antigen polymerase (Wzy) was confirmed in *L. interrogans* serovars Canicola and Copenhageni, as knockout mutants exhibited truncated LPS structures while retaining antigenic epitopes. Notably, Canicola mutants failed to induce acute disease but were recoverable from hamster renal tissue, indicating that a fully assembled LPS is critical for acute disease, while partial LPS structures may suffice for renal colonization.

## Supplementary Information

Below is the link to the electronic supplementary material.

Supplementary Material 1

Supplementary Material 2

## Data Availability

The original contributions presented in the study are included in the article/Supplementary material, further inquiries can be directed to the corresponding authors.
