# Supplementary material for: CRISPR-based mutagenesis of lipopolysaccharide biosynthesis genes in Leptospira interrogans reveals gene essentiality and confirms the role of an O-antigen polymerase
Source: Sci Rep. 2026 Mar 13;16:13419. doi: 10.1038/s41598-026-43869-y (PMC13111663; doi:10.1038/s41598-026-43869-y)
Supplement: Supplementary file 1 — Supplementary Material 1 [file 41598_2026_43869_MOESM1_ESM.docx]

**Supplementary Table 1.1- Primers used in this study**

| **Primer** | **Sequence (5’🡪3’)** |
| --- | --- |
| PEgRNA-F | ACCGCGGTGGCGGCCGAACAAGAAAGAGTCAGAGAATT |
| PEgRNA-R | CTGCAAACCGCGGCCCGAAAAGCTTTTTTTGGCTGCA |
| sgRNA-F | TTAGGATCCCCCGGGGAACAAGAAAGAGTCAGAG |
| PEgRNA-sgRNA-R | ATCGAATTCCTGCAGAAAAAAGCACCGACTCGGTGCCAC |
| LIC12137sequencing-F | ACTACCGCAATTGTATTTGGG |
| LIC12137sequencing-R | TTAGAAGGCAGCAAAGAGCCG |
| LIC12136sequencing-F | CCTATACTTTACGTGTTTAAGGCG |
| LIC12136sequencing-R | GGAATCGAATAGATTTTCCACAATCG |
| LIC12143sequencing-F | GGTTGAATCCTACGAGCGAG |
| LIC12143sequencing-R | CGTTAGATACCCACGATTTCCGG |
| LIC12135sequencing-F | GGTGTGTTTGCTTTTATATGGG |
| LIC12135sequencing-R | CGGATCAGACAAAAATGTCATCGG |
| LIC11753sequencing-F | GCTCTAAACGTGTTGTTTTGGG |
| LIC11753sequencing-R | AGTATGATCCCGTAAATTCCTGC |
| LIC_RS09320sequencing-F | AAACAATCTGAGTCCGGCG |
| LIC_RS09320sequencing-R | GAATCCAATCTATAAAAAGTCCGGG |
| LIC11312sequencing-F | GTCTGATTCCTTTGGATAAGAAGG |
| LIC11312sequencing-R | CGGTTTCCTTAAAAATACGTTCCCC |

**Supplementary Table 1.2- Protospacers for target genes**

| **Target** | **Protospacer sequence (5’🡪3’)** | **RTT+PBS (5’🡪3’)** |
| --- | --- | --- |
| LIC12137 (LIC_RS10910) | GGGTGAAGCTTTGGAGTTTA | CATGAGTTTCTAAACTCCAAAGCTTC |
| LIC12136 (LIC_RS10905) | AGGTAGCATACTAGCCAAAA | CTGGAATTAATTTTGGCTAGTATGCT |
| LIC12143 (LIC_RS10945) | TCTGAATGGCCATTACAGAG | ATAAACCAGCCTCTGTAATGGCCATT |
| LIC12135 (LIC_RS10900) | ATGATGGGAGACGAAGCGGT | GTATATACTAACCGCTTCGTCTCCCA |
| LIC11753 (LIC_RS08945) | TTGGAAGTTTACACATCCAA | CAATTTGGCATTGGATGTGTAAACTT |
| LIC_RS09320 | ATTGGAAAGTTTGTAGCAAA | TATTTAAACGTTTGCTACAAACTTTC |
| LIC11312 (LIC_RS06750) | AAGAATTGGATACGAAACAG | AAGAAAAACGCTGTTTCGTATCCAAT |
