# Supplementary material for: CRISPR-based mutagenesis of lipopolysaccharide biosynthesis genes in Leptospira interrogans reveals gene essentiality and confirms the role of an O-antigen polymerase
Source: Sci Rep. 2026 Mar 13;16:13419. doi: 10.1038/s41598-026-43869-y (PMC13111663; doi:10.1038/s41598-026-43869-y)
Supplement: Supplementary file 2 — Supplementary Material 2 [file 41598_2026_43869_MOESM2_ESM.pdf]

SUPPLEMENTARY FIGURES FOR

CRISPR-based mutagenesis of lipopolysaccharide biosynthesis genes in *Leptospira interrogans* reveals gene essentiality and confirms the role of an O-antigen polymerase

Fernandes, L.G.V.[1] and Nally, J.E[1].

[1]Infectious Bacterial Diseases Research Unit, USDA Agricultural Research Service, National Animal Disease Center, Ames, IA, United States.

Figure 1B

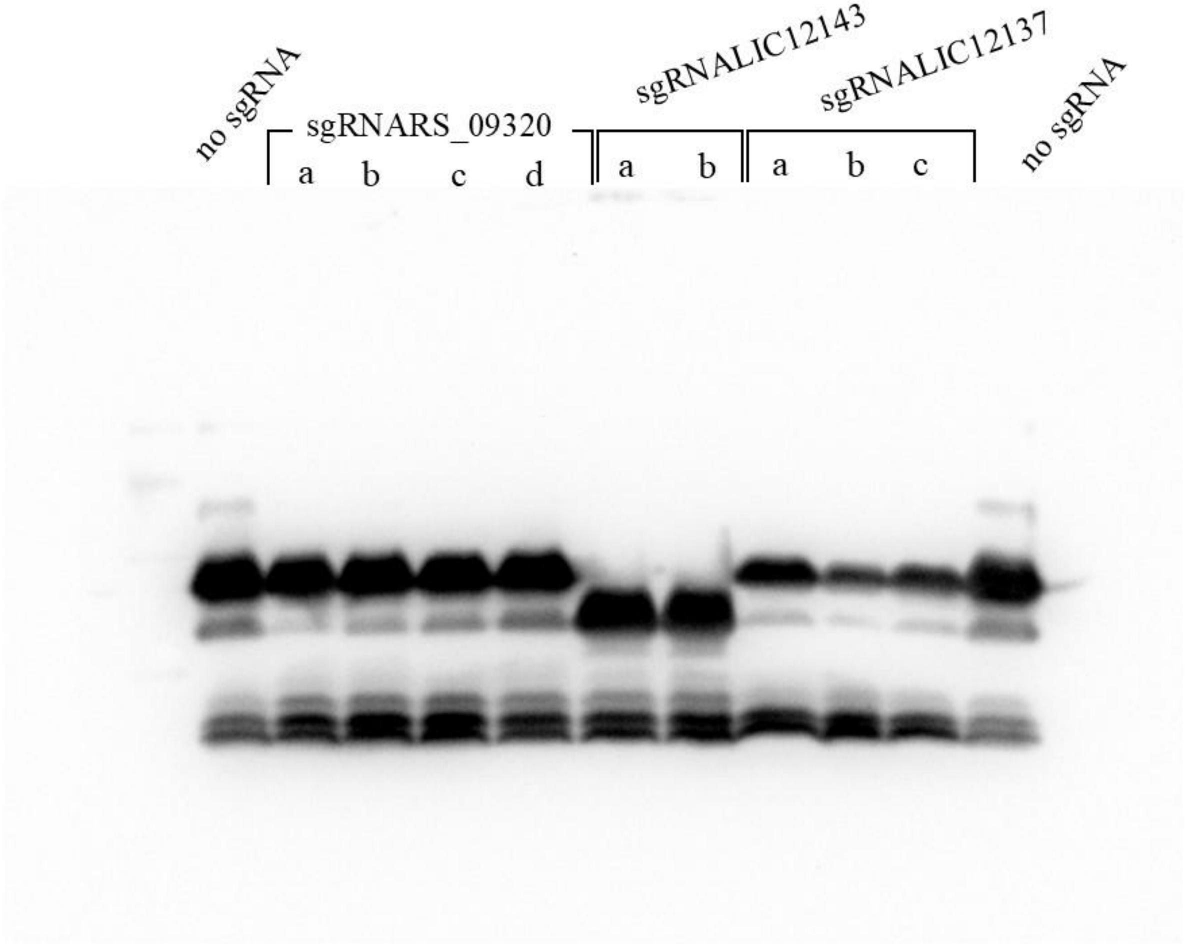

Supplementary Figure 1. Whole immunoblotting for Figure 1B. Whole-cell lysates from *L. interrogans* serovar Canicola strain LAD-1 containing pMaOriNHEJ.Inducible:Cas9, either without sgRNA or with sgRNA cassettes targeting specific genes, were analyzed by immunoblotting using anti-Canicola polyclonal reference serum.

Figure 2A

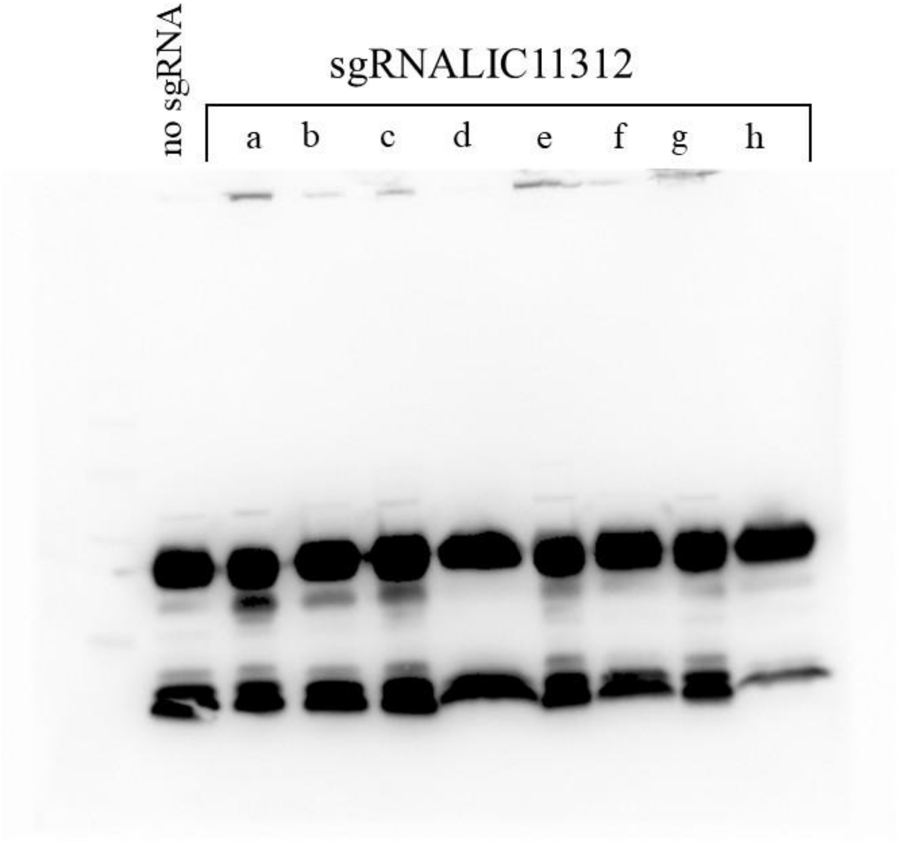

Supplementary Figure 2. Whole immunoblotting for Figure 2A. Whole-cell lysates from *L. interrogans* serovar Canicola strain LAD-1 containing pMaOriNHEJ.Inducible:Cas9, either without sgRNA or with sgRNA targeting LIC11312 were analyzed by immunoblotting using anti-Canicola polyclonal reference serum.

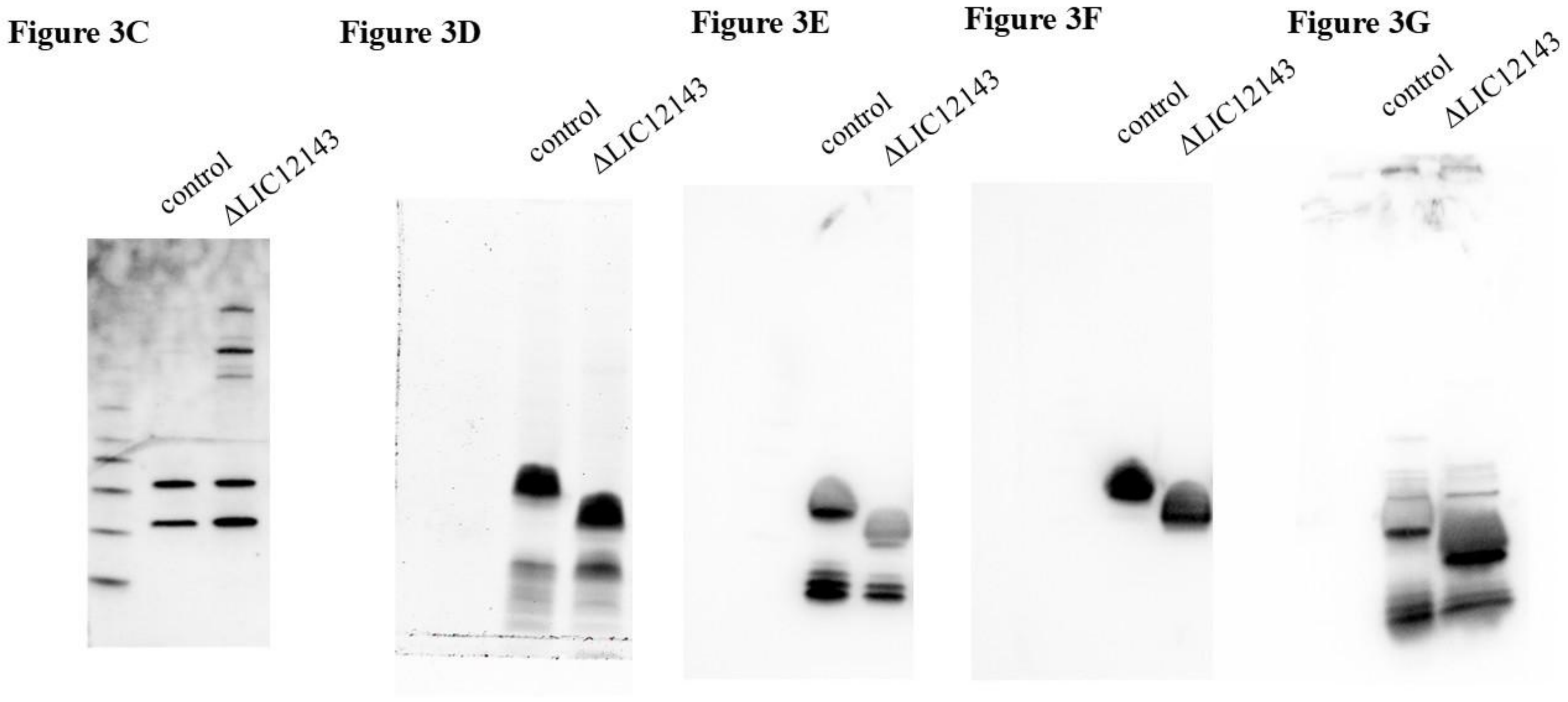

Supplementary Figure 3. Whole immunoblotting and LPS staining presented in Figure 3. Control and LIC12143 knockout (*ΔLIC12143*) *L. interrogans* serovar Canicola LAD-1 cells were analyzed by immunoblotting using anti-LipL32, anti-LipL41 (1:10,000), and anti-LigAB (1:4,000) antisera **(Figure 3C).** LPS content and molecular size were assessed by Pro-Q Emerald 300 staining **(Figure 3D)** and further evaluated by immunoblotting with anti-Canicola polyclonal (**E**) and monoclonal (**F**) antibodies. The LIC12143 gene was also disrupted in *L. interrogans* serovar Copenhageni strain R47 **(G)**, and both wild-type and mutant cells were analyzed by immunoblotting using anti-Icterohaemorrhagiae serum.

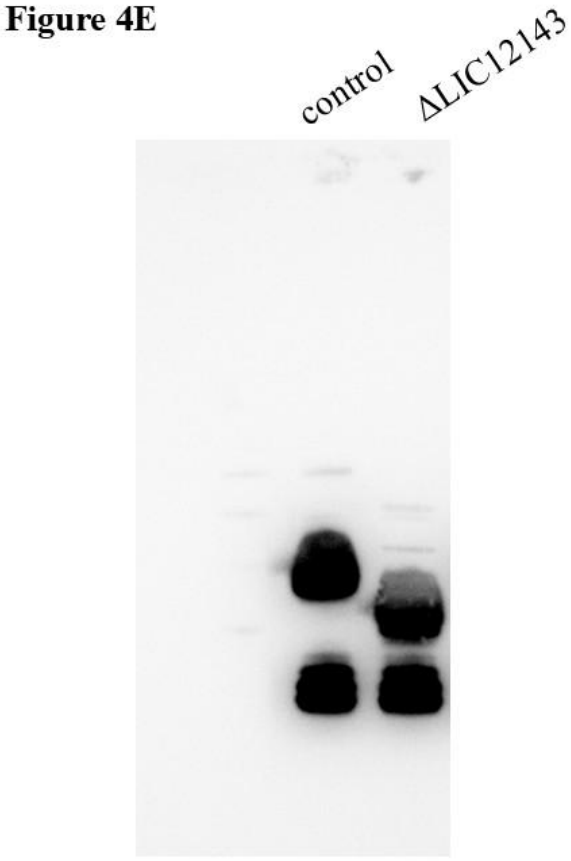

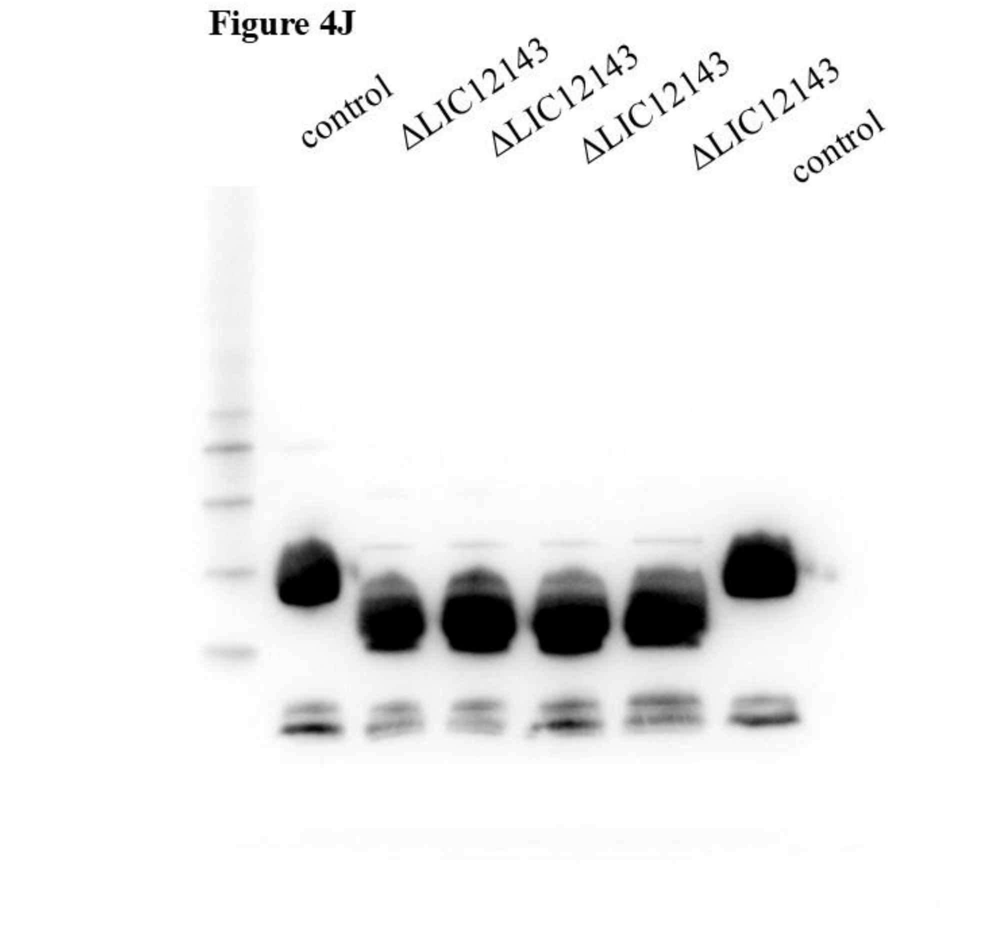

Supplementary Figure 4. Whole immunoblotting presented in Figure 4. Control and mutant LAD-1 cells isolated from kidney of infected hamsters from the first and second challenge experiments were confirmed by immunoblotting with anti-Canicola polyclonal antiserum. For Figure 4J, only one representative control and mutant lysate is presented.

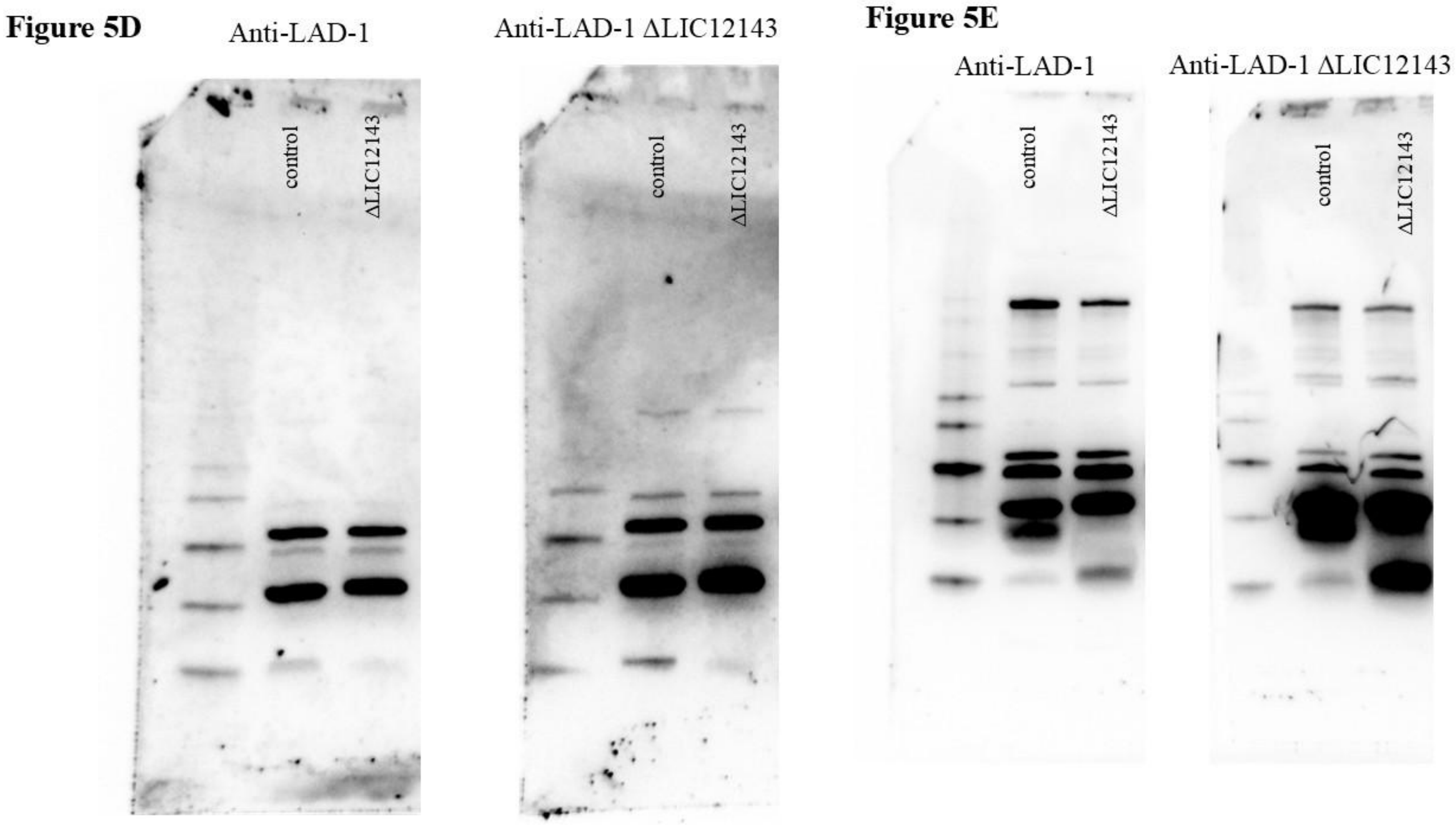

Supplementary Figure 5. Whole immunoblotting presented in Figure 5. Antibody reactivity was assessed by immunoblotting using pooled sera from vaccinated animals after primary (**D**, 1:500 dilution) and booster (**E**, 1:2,000 dilution) immunizations against lysates from both control and mutant strains. Membranes were exposed for 15 sec each
